# γδ T cells in immunotherapies for B-cell malignancies

**DOI:** 10.3389/fimmu.2023.1200003

**Published:** 2023-06-22

**Authors:** Léa Rimailho, Carla Faria, Marcin Domagala, Camille Laurent, Christine Bezombes, Mary Poupot

**Affiliations:** ^1^ Cancer Research Center of Toulouse (CRCT), UMR1037 Inserm-Univ. Toulouse III Paul Sabatier-ERL5294 CNRS, Toulouse, France; ^2^ Department of Pathology, Institut Universitaire du Cancer de Toulouse - Oncopôle, Toulouse, France

**Keywords:** immunotherapy, γδ T cells, lymphoma, leukemia, myeloma

## Abstract

Despite the advancements in therapy for B cell malignancies and the increase in long–term survival of patients, almost half of them lead to relapse. Combinations of chemotherapy and monoclonal antibodies such as anti-CD20 leads to mixed outcomes. Recent developments in immune cell-based therapies are showing many encouraging results. γδ T cells, with their potential of functional plasticity and their anti-tumoral properties, emerged as good candidates for cancer immunotherapies. The representation and the diversity of γδ T cells in tissues and in the blood, in physiological conditions or in B-cell malignancies such as B cell lymphoma, chronic lymphoblastic leukemia or multiple myeloma, provides the possibility to manipulate them with immunotherapeutic approaches for these patients. In this review, we summarized several strategies based on the activation and tumor-targeting of γδ T cells, optimization of expansion protocols, and development of gene-modified γδ T cells, using combinations of antibodies and therapeutic drugs and adoptive cell therapy with autologous or allogenic γδ T cells following potential genetic modifications.

## 1 Introduction

T lymphocytes play a critical role in anti-tumor immunity. Besides broadly discussed conventional αβ T lymphocytes, γδ T cells are also now recognized in the context of cancer inhibition. In the blood, among peripheral mononuclear cells (PBMC), γδ T cells generally account for 1 to 5% whereas they are predominant in tissues such as skin and intestine (1). Both residents, as well as circulating γδ T cells upon migration to the tumor site, can display an anti-tumor effect. With a structural difference between the γ and δ chains, γδ T cells can be divided into three main groups, Vδ1, Vδ2 and Vδ3 T cells, all of which recognize antigens independently of the major histocompatibility complex (MHC) molecules.

In B-cell malignancies, such as B-cell lymphomas, chronic lymphocytic leukemia (CLL) or multiple myeloma (MM), tumor cells can be found both in peripheral blood (PB) and in lymphoid organs, such as bone marrow (BM) or lymph nodes (LN). Therefore, these malignant cells can interact with other cell types constituting a specific microenvironment, in which infiltrating γδ T cells can play an important role. Vδ1 and Vδ2 T cells have been described to participate in the anti-cancer responses in B-cell malignancies with sometimes different proportions and different modes of action.

In this review, we first described γδ T cell diversity in B-cell lymphomas, CLL and MM. We then focused on γδ T cell activation and finally we presented attractive candidates for immunotherapies (IT) in B-cell malignancies.

## 2 γδ T cell diversity in B-cell lymphomas, CLL and multiple myeloma

Following T cell receptor (TCR) rearrangement, γδ T cells can be categorized into three main groups: the variable Vγ9 chain paired with Vδ2 (Vγ9Vδ2 T cells, also known as Vδ2 cells) (2), the variable Vδ1 chain with different Vγ chains (3) and Vδ3 T cells. Lymphocytes expressing heterodimers of Vδ2 and Vγ9 chains are predominant in the blood where they account for most (50–95%) of the γδ T cells, whereas Vδ1 T cells (paired with various Vγ chains) are more abundant in tissues, including healthy epithelia or solid tumors (4). Vδ3 like Vδ1 T cells were shown as dominant in the intestinal mucosa, skin, and liver (3), and to actively participate in cancer immunobiology.

These lymphocytes can differentiate into different T helper-like cells (Th1-, Th2-, Th9-, and Th17-like cells), producing a wide range of cytokines to fulfill their physiological role (5–7). More precisely, γδ T cells can harbor different phenotypes, such as: naive, central memory (CM), effector memory (EM) or RA^+^ effector memory (TEMRA) (8, 9). Moreover, γδ T cells co-express other functional receptors, including activating natural killer receptors (NKR: NKG2D, NKp30 and NKp44) (10, 11) and various Toll-like receptors (TLRs) (12). However, they can also express inhibitory NKR such as CD94/NKG2A or immune checkpoints (ICP), such as: PD-1, TIM3, LAG3 or CD39. Interestingly, NKG2A^+^ Vδ2 T cells were shown to exert higher anti-tumor potential (13).

Patients with Hodgkin’s Lymphoma (HL) were characterized with a marginally higher level of circulating γδ T cells, compared to healthy donors (14). The tumor escape from immune surveillance by the γδ T cells in these patients could therefore be due to the immunosuppressive profile of these cells plus an increase of soluble MICA derived from its shedding at the surface of lymphoma cells. Interestingly, HIV-infected individuals developing HL were also shown to display a significant expansion of the Vδ1 T cell subset compared to those without HL. To go further, the authors showed a high expression of CD16 and the inhibitory receptor CD158b by these Vδ1 T cells, concomitantly with a low expression of CCR5, CXCR4 and CXCR3, thus decreasing their homing to the tumor site (15). This discrepancy could point to a causal role in the pathogenesis of HL.

On the other hand, in B-cell non-Hodgkin’s lymphomas (B-NHL), the major subtypes of circulating γδ T cells were shown to be Vγ1, Vδ1 and Vδ2 (16). Compared to healthy donors, patients exhibit an absence of Vγ2 TCR subfamily in PB, BM, and LN. This implies a widespread restriction of the Vγ gene expression repertoire that may be a feature in patients with B-NHL. Moreover, the distribution of Vγ and Vδ subfamilies varied between PB, BM, or LN, and this may be due to the distribution or expansion of γδ T cells in different immune organs and to local immune responses (16).

In the case of diffuse large B-cell lymphoma (DLBCL), an aggressive form of B-NHL, γδ T cells represent a substantial population among infiltrating T lymphocytes. Amongst this population, Vδ1 T cells were shown as the major γδ T cell subset in both tumor and PBMC, whilst Vδ2 T cells were the most common subset in PBMC of healthy donors (17). In this study, the Vδ1 T displaying a naive phenotype (whether in blood or in LN) were shown as functional, however the authors did not observe any correlation between the rate of Vδ1 T and well-established prognostic factors, clinical responses or progression-free survival (PFS). Interestingly, the germinal center (GC) subtype of DLBCL was associated with an increase in Vδ1 cells in the tumors, whereas the non-GC subtype was associated with a lower frequency of γδ T cells (17). Activation or reactivation of Vδ1 T cells in DLBCL patients either by using *ex-vivo* expanded cells or by promoting their expansion *in vivo*, could represent a therapeutic outcome.

In the case of follicular lymphoma (FL), Braza and collaborators showed that γδ T cells as well as CD8^+^ T lymphocytes were located in the perifollicular zone of the LN of FL patients and not inside follicles. The majority of FL-LN γδ T cells are Vδ2 CCR7^+^ unlike circulating ones, whereas expression of the chemoattractant CCL19 chemokine is lower in FL-LN than in inflamed LN, explaining the low γδ T cell count in FL-LN (18). However, γδ T cells from FL patients displaying good cytolytic properties against lymphoma cells, *ex-vivo* expansion or promotion of *in vivo* expansion could be a therapeutic option if expanded γδ T cells can home in to the tumor site. Nevertheless, in this study, the authors considered only the Vδ2 subtype without taking into account the Vδ1 cells, which can counterbalance the decrease of Vδ2 T cells. In another study, the authors compared reactive LN from lymphoma-free individuals with FL-LN. Unlike Braza’s work, they showed no significant difference in the percentage of the cytolytic γδ T cell population between reactive LN and FL-LN (19). The immune microenvironment of LN can therefore have an important impact on the phenotypical and functional characteristics of infiltrated T cells.

Increase in Vδ1 T cells was also observed in CLL and MM patients. The analysis of PB of patients with CLL revealed a general prevalence of the Vδ1 T cell subtype, with an increased cell count in more severe stages of the disease (20). This increasing percentage of Vδ1 cells was also observed as belonging to the CD27^-^ compartment from controls to advanced stages of CLL patients, in particular in Binet B and C CLL groups, exhibiting a cytotoxic phenotype with the expression of granzyme B (11). Another study showed an increase of Vδ1 cells in the blood of CLL patients with stable disease, which were able to proliferate and produce TNF-α and IFN-γ in response to autologous CLL cells suggesting the potential of Vδ1 cells based therapies in this disease (21). These contradictions could be explained by the expression of some exhaustion markers, not determined in this study which can thwart the cytotoxic efficacy. An increase of exhaustion markers such as PD-1, TIGIT, TIM3 and CD39, expressed by Vδ1 cells was also shown in BM of MM patients. Whilst these patients displayed no difference regarding an overall level of γδ T cells in comparison to healthy donors, they showed a higher proportion of Vδ1 over Vδ2 T cells (22). Elevated percentage of γδ T cells with an exhausted phenotype (PD-1^+^), associated with a decreased expression of genes involved in effector functions was also found in patients with relapsed/refractory MM (23). However, exhaustion problems could be overcome by using ICP inhibitors such as anti-PD-1 antibodies. Additionally, inhibition of the anti-tumor immune response was associated with elevated levels of γδ regulatory T cells in the PB of MM patients with a bad prognosis (24) as well as in CLL patients (25). Besides, the increase of circulating cytotoxic γδ T in PB of MM patients after autologous hematopoietic stem cell transplantation was associated with improved PFS and overall survival (OS) (26).

Finally, γδ T cells were detected in all B-cell malignancies with an exhausted phenotype. Their reactivation or manipulation with immunotherapeutic approaches could represent a promising therapeutic option for patients with these diseases as developed in the section 4.

## 3 γδ T cell activation in B-cell lymphomas, CLL and multiple myeloma

**Figure 1:**
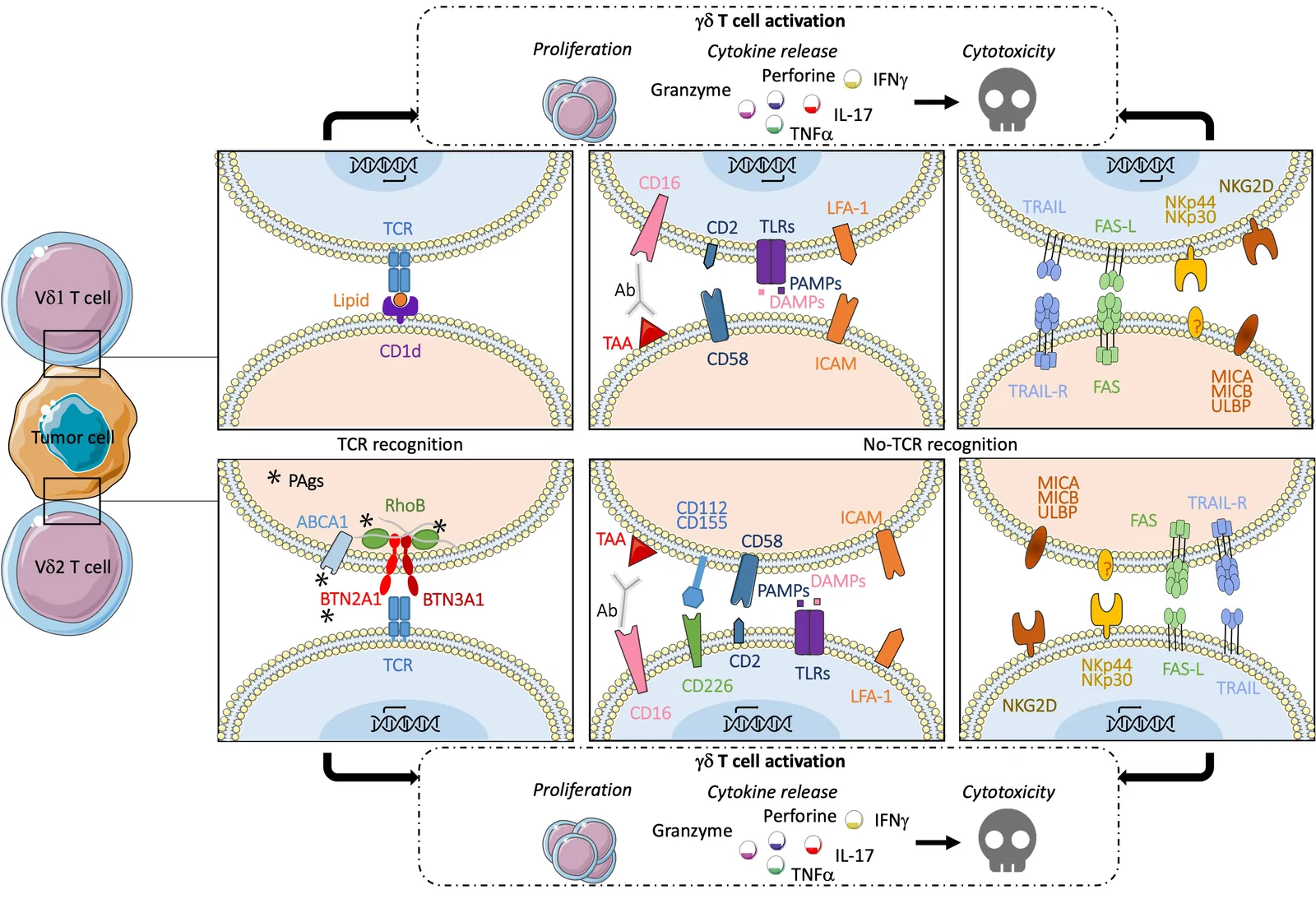
γδ T cell activation through different TCR-dependent and TCR-independent pathways leading to proliferation and/or cytokine release and/or cytotoxic signals.

Depending on their TCR variant, γδ T cells, can respond to a variety of antigens. Vδ1 and Vδ3 cells can recognize, *via* their bound TCR glycolipids, MHC-related class Ib molecules CD1c for Vδ1 (27) and CD1d for Vδ3 (28). On the other hand, Vδ2 cells recognize non-peptidic antigens in the form of small pyrophosphate molecules called phosphoantigens (PAg), that can be found endogenously, such as hydroxymethyl-butyl-pyrophosphate (HMBPP/HDMAPP) or metabolites of the mevalonate pathway, or synthetic molecules, such as BrHPP (bromohydrine pyrophosphate) (29, 30). Zoledronate, a third generation aminobisphosphonate, a farnesyl pyrophosphate synthase inhibitor, widely used for osteolysis, has been shown to enhance antitumor Vδ2 cell responses, through the overexpression of endogenous PAg by tumor cells (31, 32). MHC-class I molecules are not involved in PAg recognition by Vδ2 T cells, but other molecules including butyrophilins (BTN3A1/BTN2A1), the ABCA1 transporter, the intracellular RHOB or periplakin molecules were shown to be involved in their activation (33–37). Besides TCR involvement, γδ T cells expressing NKR and TLRs, are also able to respond to stress-induced NKR ligands such as the ribonucleic acid export 1 (RAE1), MHC class I-related molecule A or B (MICA/MICB), UL16-binding proteins (ULBPs) (11, 38) amongst other DAMPs or PAMPs (12). Interaction of γδ T cells with cancer cells expressing these molecules, leads to the formation of an immunological synapse (39, 40) resulting in γδ T cell proliferation, cytokine release and tumor cell lysis (41). However, other molecules such as CD226 (DNAX accessory molecule-1), adhesion molecules (LFA-1), CD3 or CD2, can also be involved in γδT cell activation and favor their immune responses. These different activation modes are summarized in **Figure 1**.

Considering the maturation stages, γδ TEM cells expressing high levels of chemokine receptors, produce large amounts of IFN-γ and TNF-α in response to TCR stimulation and γδ TEMRA cells, which express several NKR but low levels of chemokine receptors, are highly active against tumoral target cells and efficient to ADCC thanks to CD16 expression. These γδ T effector cells express their cytotoxicity through the production of high amounts of perforin and granzyme. Naive and CM cells do not display effector functions but are able to proliferate.

γδ T cells from patients with B-cell malignancies and particularly Vδ2 T cells were evaluated for their functionality, by the *in vitro* sensitization of cancer cells or cells of the tumor microenvironment with zoledronate (31, 32). In NHL, LN mesenchymal stromal cells were shown to interfere with Vδ2 lymphocyte cytolytic function and differentiation into Th-1 or EM cells but pre-treatment of these immunosuppressive cells with zoledronate can rescue lymphoma cell killing *via* the TCR and NKG2D (32). Vδ2 T cells from patients with B-cell lymphoma and MM, expanded *in vitro* by culture with zoledronate and IL-2, displayed enhanced cytotoxic effects towards MM/B-cell lymphoma cell lines and autologous tumor cells, without cytotoxicity against normal cells in these patients (31). However, approximately 50% of untreated MM patients showed Vδ2 T cells that were unable to proliferate upon stimulation with zoledronate and IL-2, but had strong effector properties exhibiting TEM or TEMRA phenotypes (42, 43). Similar results have been described for untreated CLL patients, who were classified as responders and displayed proliferation of zoledronate-stimulated Vδ2 T cells (25). Interestingly, the low-responders showed significantly greater baseline peripheral Vδ2 T cell counts than the responders, ruling out a quantitative defect. Indeed, the low-responder patients showed an accumulation of TEM and TEMRA Vδ2 cells with high effector functions and low capacity to proliferate, whereas naive and CM were preferentially found in responding patients (25, 44). In addition, Coscia and collaborators showed that a low proliferation capacity of Vδ2 cells was correlated with subsets of CLL patients with unmutated immunoglobulin heavy variable (IGHV) genes (U-CLL). This is in agreement with the upregulation of NKG2D on γδ T cells of CLL patients responding to zoledronate (25). Vδ2 T cells isolated from PBMC of MM patients were also shown to upregulate NKG2D upon *in vitro* expansion with zoledronate. Additionally, a low-dose treatment with bortezomib (proteasome inhibitor typically used in MM) sensitized MM cells to *in vitro* lysis by Vδ2 cells through NKG2D (45). Vδ1 cells may also be involved in the anti-cancer response towards CLL cells through NKG2D activation. Indeed, Vδ1 cells, which are enriched in PB of CLL patients, can kill neoplastic CLL cell lines transfected with MICA, and blocking anti-NKG2D antibody largely decreases autologous leukemic cell lysis (21). In addition, purified Vδ1 cells isolated from the PB of MM patients can kill MM cell lines, and produce cytokines involving their TCR and NKG2D, DNAM-1 and adhesion molecules (46). Unfortunately, cancer cells are able to express some enzymes such as ADAM 10 and 17 and are able to shed the stress molecules MIC-A and–B and ULBPs from their surface, therefore decreasing the γδ T response through NKG2D (47).

As γδ T cells, whether Vδ1 or Vδ2, can be activated by B-cell lymphoma, CLL or MM cells, these cells represent essential actors in anti-tumor responses against B-cell malignancies and can definitely be good targets for IT in these diseases.

## 4 γδ T cell-based immunotherapies in B-cell malignancies

For a long time neglected, γδ T cells have emerged as a key immune cell type in cancer biology, representing very attractive and promising candidates for cancer IT. Their therapeutic potential in solid and hematological cancers have been extensively reviewed elsewhere (48–53) and here we focused on their exploitation in B-cell malignancies.

**Figure 2:**
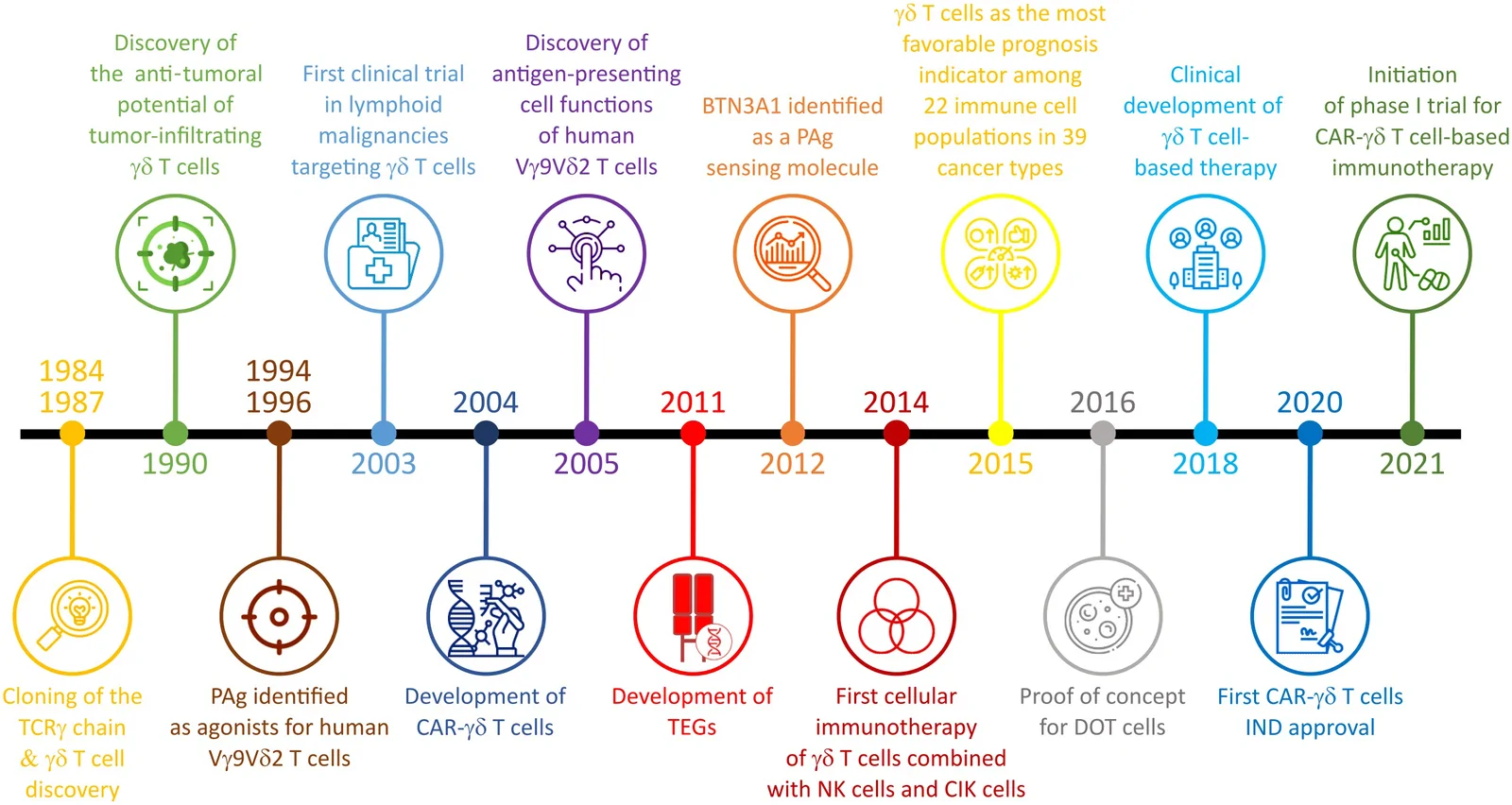
Discovery timeline of the γδ T cell role in cancer and γδ T cell-based IT (adapted from *Silvia-Santos et al., Nature Review 2019* and *Bhat et al., Frontiers in Immunology 2022*).

γδ T cells can be used in several strategies, based on the *in vivo* activation to potentiate the tumor-targeting or the optimization of *in vivo* or *ex vivo* expansion protocols. These approaches consist of: i) combination with therapeutic drugs or antibodies, ii) adoptive cell transfer (ACT) using of autologous or allogenic γδ T cells expanded *ex vivo* and iii) ACT using genetically modified γδ T cells. The timeline of γδ T cell-based IT is presented in the **Figure 2** and the various cell-based IT, ongoing clinical trials, and associated sponsors are summarized in the **Table 1**.

**Table 1:** Summary of γδ T cell-based IT, ongoing clinical trials and associated sponsors.

Cell-Based Immunotherapy approaches in B-cell malignancies							
Type of therapy	Disease		Agents in development			Sponsors	Reference/ Clinical trial number
Combination with therapeutic drugs or antibodies							
Zoledronate/IL-2	MM		NA			NA	(60)
Pamidronate/IL-2	Relapsed/refractory low-grade NHL and MM		NA			NA	(61)
BrHPP/IL-2	FL		NA			NA	(63)
Zoledronate	ALL and AML		NA			NA	(65, 66)
Zoledronate/IL-2	Hematological malignancies		NA			Nantes University Hospital	NCT03862833
Anti-BTN3A1+anti-PD-1	R/R DLBCL and FL		ICT01			ImCheck Therapeutics	NCT04243499
vγ9TCRxCD1d bAb	R/R CLL, AML and MM		LAVA-051			Lava Therapeutics	NCT04887259 (74),
vγ9TCRxCD40 bAb	CLL, MM		LAVA-1278			Lava Therapeutics	(75)
CD19xCD16bAb	ALL		NA			NA	(73)
Adoptive cell transfer using of autologous							
Zoledronate-activated Vδ2 T cells	CLL, AML, ALL					University of Kanas Medical Center & In8bio Inc.	NCT03533816
Adoptive cell transfer using allogenic γδ T cells expanded ex vivo							
Zoledronate/IL-2 activated allogeneic γδ T cells from healthy donors	Advanced refractory MM						(83)
2 cycles of 2 dosage escalated manner infusions at 14 days intervals	R/R NHL					Institute of Hematology & Blood Diseases Hospital & Beijing GD Initiative Cell Therapy Technology Co	NCT04696705
Dose escalation between 3 cohorts (negative MRD or SD, positive MRD but not HR, HR)	AML, ALL, Lymphoma					Chinese PLA General Hospital & Beijing	NCT04764513
Allogenic vd1 and T cell therapy	AML, CLL		GDX012			GammaDelta Therapeutics Limited	NCT05001451, (84, 97)
Adoptive cell transfer using γδ T cells genetically modified							
CD20 directed CAR-δ1 T cells	FL, MCL, MZL, DLBCL, NHL		ADI-001			Adicet Bio, Inc	NCT04735471 (89, 90),
αβ T cell product retrovirally transduced with vγ9vδ2 TCRs	AMLR/R MM		TEG001TEG002			Gadeta B.V.	NCT04688853, (93, 94)
CD19-CAR (Ab)TCR	R/R CD19^+^NHL		ET190L1 ARTEMIS™			Duke University & Duke Clinical Research Institute Peking University & Eureka(Beijing) Biotechnology	NCT03379493, NCT03415399 (95),
CD19-directed CARvγ9vδ2 T cells	ALL		NA				(86)
Anti-CD19 CAR- γδT cells	R/R CD19^+^ B-cell leukemia and lymphoma		ET019003-T Cells			Wuhan Union Hospital, China &Eureka(Beijing) Biotechnology	NCT04014894

NA, not applicable.

### 4.1 Combination of γδ T cells with therapeutic drugs or antibodies

γδ T cells can be directly activated by drugs or antibodies modifying their effector properties and/or potentializing their *in vivo* expansion, but also indirectly by increasing the sensitization of cancer cells.

Concerning sensitization of tumor cells, Poggi and collaborators showed that trans-retinoic acid, an active metabolite of vitamin A, was able to induce MICA expression at the surface of CLL cells from patients, leading to an increase of their lysis by autologous Vδ1 T cells (21). Other drugs such as bortezomib, were also involved in the up-regulation of NKG2D and DNAM-1 ligand expression by MM cells, leading to the enhancement of the Vδ2 T cell cytotoxic effect (45). Moreover, the use of ADAM 10 and 17 inhibitors on HL cell lines revealed an increase of their sensitivity to NKG2D-dependent cell killing mediated by NK and γδ T cells (47). Another class of molecule, HDAC inhibitors, were also able to increase expression of NKG2D ligands by pancreatic or prostate cancer cells (54) and could be interesting in B-cell malignancy therapies. However, these molecules also suppress the γδ T cell anti-tumor functions inducing a non-functional truncated form of NKG2D and increasing ICP expression (55, 56). Another way to increase the NKG2D mediated anti-tumor effect of γδ T cells, is to use recombinant immunoligands consisting of a CD20 single-chain fragment variable (scFv) linked to MICA or ULBP2. Indeed, killing by both Vδ2 and Vδ1 T cells, of CLL cells from patients and lymphoma cell lines sensitized by these two immunoligands was significantly increased (57).

The direct activation of γδ T cells can be achieved by the TCR dependent pathway through exogenous or endogenous PAg. As mentioned previously, aminobisphophonates induce the production of endogenous PAg in tumor cells. Treatment of BM mononuclear cells from MM patients with aminobisphophonates induced an *in vitro* stimulation of γδ T cell-mediated anti-plasma cell activity, as well as a tumor regression in myeloma xenografted mouse models (58, 59). However, a phase II clinical trial of zoledronate/IL-2 treated MM patients after BM transplantation led to only 18% of complete remission (CR) due to a progressive reduction of γδ T cells *in vivo* expansion despite several cycles of zoledronate/IL-2 injections (60).

In addition, an *in vivo* amplification of autologous γδ T cells has been shown following injection of aminobisphosphonates and IL-2. The first study was conducted by Wilhelm and collaborators, where a low-dose of IL-2 in combination with pamidronate was tested, according to two different schedules, in 19 patients with relapsed/refractory low-grade NHL (FL, CLL, mantle cell lymphoma-MCL) and MM (61). The first treatment schedule consisted of administration of pamidronate on day 1 followed by increasing dose levels of IL-2 from day 3 to day 8. Unfortunately, only 1 out of 10 treated patients achieved a stable disease. The other treatment schedule consisted of pamidronate infusion, followed directly by IL-2 administration from day 1 to day 6. In that case, a significant *in vivo* activation/proliferation of γδ T cells was observed in 5 out of 9 patients and objective responses were achieved in 3 patients (33%). Interestingly, *in vivo* proliferation of γδ T cells was associated with tumor regression confirming a γδ T cell-mediated anti-lymphoma effect. This correlation was also observed in a B-cell depletion assay from cynomolgus monkeys injected with the regimen combining an anti-CD20 mAb (rituximab) with BrHPP and IL2 (62). Thanks to promising results from pre-clinical studies (18), it was possible to enter clinical phase I/II studies where the effect of BrHPP (IPH1101) combined with low doses of IL2 was evaluated in 45 FL patients. The treatment induced a strong and specific amplification of γδ T cells with a 45% overall response rate (63). Unfortunately, the final outcomes were never published. Besides, Zoledronate exhibited promising results in hematological malignancies, such as acute myeloid leukemia (AML) (64) where 25% of partial response was reached. In pediatric acute lymphocytic leukemia (ALL) and AML, after B- and αβ T-cell-depleted and HLA-haploidentical hematopoietic stem cell transplantation (HSCT), infusion of zoledronate lowered transplantation-related mortality, increased the number of circulating γδ T cells and improved disease-free survival (65, 66). Currently, a phase I clinical trial is open to determine the maximum tolerated dose of early administration of increasing doses of IL-2 in combination with a fixed dose of zoledronate, in order to expand Vδ2 cells in patients with a hematological disease eligible for a haplo-stem cell transplantation (NCT03862833).

Some antibodies have also been shown as activating γδ T cell anti-tumor functions. A first-in-class humanized anti-BTN3A1 antibody was designed to harness and enhance Vδ2 cell–driven anti-tumor activity against multiple tumor cell lines and primary tumor cells (67), opening promising perspectives. A phase I/IIa trial in patients with advanced-stage relapsed and/or refractory cancers including DLBCL and FL (NCT04243499) are currently opened to assess the safety, tolerability and efficacy alone or in combination with the anti-PD-1 mAb pembrolizumab.

ICP blocking antibodies can also favor the anti-tumor cytotoxic potential of γδ T cells, against the Burkitt lymphoma cell line Raji for instance (55), arguing that in some cases, ICP can be the only barrier to the cytotoxic functionality of these effector cells. These antibodies in MM treatment were shown to improve PAg-activation of Vδ2 T cells as, in the BM microenvironment, these cells largely express PD-1 hampering their PAg-reactivity (68, 69). This anergy was also detected in CLL with a reduction of cytotoxicity related to reduced granzyme secretion (44), opening up the possibility of using anti-ICP antibodies in CLL treatment.

Antibodies targeting Fc receptors, such as CD16 expressed by γδ T cells, can also artificially enhance their cytolytic function *via* ADCC. Efficacy of ADCC in B-cell malignancies has been shown using anti-CD20 (19, 62, 70–72), anti-CD52 (62) or anti-CD38 (72) mAbs, all these antibodies target different molecules at the surface of cancer cells. Moreover, potentiation of ADCC using anti-CD20 was observed with the BrHPP/IL-2 stimulation of Vδ2 T cells from PBMC of CLL patients in autologous co-cultures (62).

Finally, another category of antibodies consists of bispecific Ab (bsAb) that simultaneously bind γδ T cells and a tumor antigen. These bsAb strongly enhanced lysis mediated by γδ T cells, as shown for the SPM-1 Ab, a single chain trispecific Ab (triplebody or tribody) directed against CD19-CD19-CD16 that efficiently redirected lysis of CD19-bearing target cells (73). De Weert and collaborators, showed a robust activation and degranulation of Vδ2 T cells in co-culture with autologous CLL cells expressing CD1d treated with vγ9TCRxCD1d bsAb (LAVA-051) (74). As CD40 is also overexpressed in CLL and MM, the bsAb CD40-Vγ9Vδ2 T cell engager (LAVA-1278) was shown as promoting a potent Vδ2 T cell degranulation and cytotoxicity against CLL and MM cells *in vitro* and *in vivo* (75). Recently, a novel bispecific molecule was developed by linking the extracellular domains of tumor-reactive Vγ9Vδ2 TCR to a CD3-binding moiety, creating γδTCR-anti-CD3 bispecific molecules (GABs). The high affinity of Vδ2 for PAg enriched in tumor cells favored the recruitment of other CD3^+^ T cells in the TME enhancing the *in vivo* targeting of MM cells and leading to tumor regression (76). Altogether, these bsAbs represent promising candidates for the development of novel treatments for B-cell malignancies and for now, only one phase I/II clinical trial is opened (NCT04887259) to assess the efficacy of LAVA-051 (vγ9TCRxCD1d bAb) in patients with relapsed/refractory CLL and MM in whom it appears to be well tolerated (77).

### 4.2 Adoptive cell transfer with autologous γδ T cells

One of the biggest advantages of using autologous γδ T cells in adoptive cell transfer for IT is the lack of graft versus host disease (GVHD). The ACT of autologous γδ T cells requires *ex vivo* expansion of γδ T cells thanks to the activation of purified PBMC from the blood of the patient by IL2 and either natural (isopentenyl pyrophosphate-IPP and HMBPP) or synthetic (BrHPP) PAgs. The prerequisite being the capacity of patient γδ T cells to be expandable in *in vitro* culture. In the case of activation with exogenous PAg added to IL-2, PBMC of CLL patients showed a significant *ex vivo* expansion of Vδ2 T cells with the ability to secrete lytic granules leading to the efficient killing of autologous CLL cells (62). *Ex vivo* expansion of γδ T cells offers an opportunity to characterize their phenotype and sort the cells with the highest anti-tumoral potency, prior to their reinfusion into patients. So far, this approach has only been described for solid tumors (78). In MM patients, high-dose administration of *ex vivo* zoledronate-activated Vδ2 T cells resulted in a measurable increase of Vδ2 cell number in PB and BM, which was correlated with an anti-tumoral effect in 4 of 6 patients (79). A phase I clinical trial is currently opened in CLL (NCT03533816) in order to extract, concentrate, and activate γδ T cells from the PB to provide an innate anti-tumor effect.

### 4.3 Adoptive cell transfer with allogenic γδ T cells

In almost all autologous studies, treatments showed the reduction of tumor burden some patients, but the effects were inconsistent. It becomes clear, that the failure of these strategies can be due to the poor T-cell fitness of patients heavily pre-treated with chemotherapy. Thus, to overcome this issue, a huge effort has been developed to propose “off-the-shelf” therapies using allogenous γδ T cells isolated from healthy donors. Due to their unique property to recognize antigen in a MHC independent manner and that they do not require HLA-matching of donors and recipients, γδ T cells are ideal candidates to develop ACT strategies. ACT with allogenic Vδ2 cells is more often tested in patients harboring solid cancers (80–82). In hematological malignancies, only one study reported the infusion of allogeneic γδ T cells from healthy donors, in patients harboring, amongst others, advanced refractory MM who were not eligible for allogeneic transplantation (83). Proliferation of γδ T cells peaked after 8 days and donor cells persisted up to 28 days. Although refractory to all prior therapies, 3 out of 4 patients achieved a CR, which lasted for 8 months in a patient with plasma cell leukemia. Thus, this pilot study indicated that the use of allogeneic γδ T cells, from selected donors who were half-matched (HLA-haploidentical) family members, is feasible and safe, and that zoledronate/IL-2 infusions can activate and expand allogeneic γδ T cells *in vivo* to achieve promising therapeutic responses.

### 4.4 Adoptive cell transfer with allogenic genetically modified γδ T cells

The Vδ1 subset of γδ T cells is a promising candidate for cancer IT but suffers from the lack of a suitable expansion/differentiation method. Thus, without any genetic modification, Sebestyen’s group was the first to develop a robust and reproducible clinical-grade method for generating cytotoxic Vδ1 T cells called Delta One T (DOT) cells that have been expanded and differentiated (84). Based on studies of CLL models, DOT exhibited cytotoxic features and specifically targeted leukemic *in vitro* and in preclinical *in vivo* models (cell line- or patient derived-xenograft), controlling the burden and dissemination of cancer cells (84).

Concerning CAR-T cells, αβ T cells were the first T cells developed for ACT. However, even though CAR-αβ T cells are still developed in cancer IT, potential GVHD apart from cytokine toxicity and antigen escape pose limitations to this approach. CAR-γδ T cells rapidly become a highly interesting alternative due to their HLA-independent antitumor immunity. Thanks to the progress made in the field of engineering and expansion protocols consistent with current good manufacturing practices, CAR-δ1 and CAR-δ2 T cells were developed during the recent years. Therefore, CAR-γδ T cells appeared to have their niche in situations where conventional CAR therapy is less suitable. In 2004, Rischer and collaborators were pioneers to demonstrate that zoledronate-activated CD19-CAR-γδ T cells exhibited a potent and specific anti-tumor activity against B cell malignancies *in vitro* (85). Ten years later, the group of LJN Cooper developed a CD19-directed CAR-γδ T cell that displayed enhanced killing of CD19^+^ tumor cells *in vitro* and in leukemia xenograft models (86). These observations were also obtained by other groups (87).

Due to their long persistence *in vivo*, Vδ1 T cells represent attractive candidates for ACT. Based on the “DOT protocol” (84), a clinically translatable protocol for Vδ1 T cell expansion allowed the development of CAR-δ1 T cells that exhibited highly consistent innate cytotoxicity against different leukemic cell lines (88). Of note, CD20 directed CAR-δ1 T cells (under the name ADI-001) exhibited a potent anti-cancer activity both *in vitro* and *in vivo*, in B-cell lymphoma xenografts in NSG mice bringing strong evidence to propose the assessment of its efficacy in phase I clinical trial (NCT04735471) in patients with B-cell malignancies (89). The first results showed that ADI-001 maintained a favorable safety profile, and preliminary efficacy showed very encouraging CR rate (4/5) and sustained durability in patients, including those previously exposed to conventional CAR-T therapy (90). Based on the same strategy, a 4-1BB-based CAR-DOT directed against CD123 was generated and preclinically validated in AML, with a potent cytotoxicity against cell lines and primary samples both *in vitro* and *in vivo*, even following a tumor rechallenge (91).

**Figure 3:**
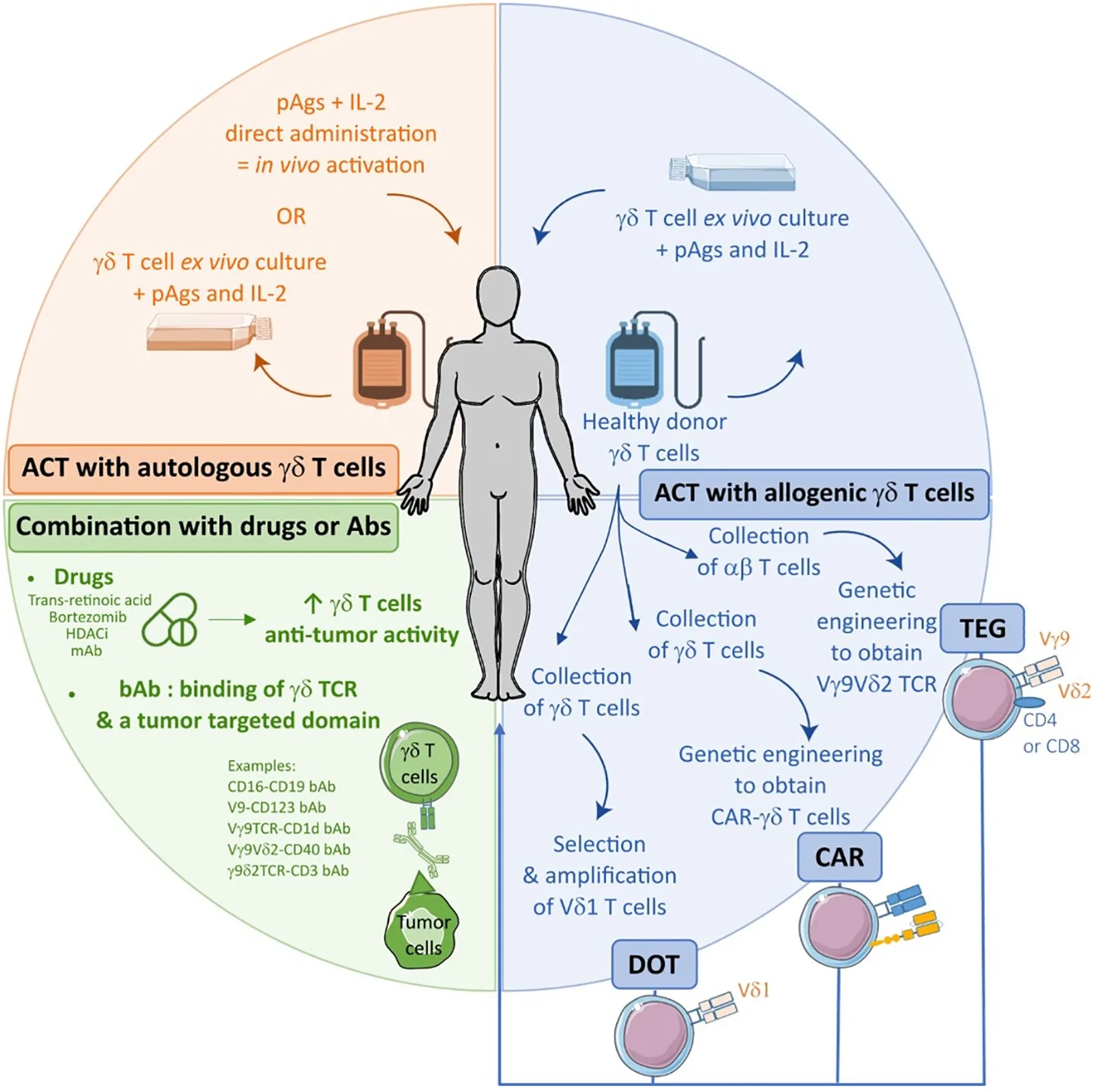
Spectrum of γδ T cells-based immunotherapies in B-cell malignancies from the use of drugs or antibodies to adoptive cell transfer (ACT) of autologous or allogenic cells.

Although promising results were obtained with CAR-γδ T cells, limited proliferative capacity of Vδ2 cells and their underestimated diversity, led to the development of αβ T cells engineered to express a defined γδ TCR, the so-called TEGs (92), that can target a broad range of hematological tumors (93, 94). Interestingly, these cells not only exhibited strong anti-tumor reactivity and potent proliferative capacity of αβ T cells, leading to tumor eradication in leukemic PDX models, they also retained both CD4^+^ and CD8^+^ effector cell functions (94). Currently, a phase I clinical trial (NCT04688853) is testing the TEG002, an autologous T cell transduced with a specific γδ TCR, in relapsed/refractory MM patients. Another strategy is based on the combination of the Fab domain of an antibody with the γ and δ chains of the TCR (AbTCR) as the effector domain (95). This CD19-CAR (Ab)TCR (ET190L1 ARTEMIS™) triggered Ag-specific cytokine production, degranulation and killing of CD19^+^ cancer cells *in vitro* and in a xenograft mouse model. Whether these pre-clinical findings for AbTCR translate into clinical settings has been assessed in two clinical trials in relapsed and refractory CD19^+^ NHL (NCT03379493, NCT03415399). Very recently, a novel anti-CD19 CAR-T cell system was obtained by fusing the anti-CD19 antibody Fab domain with the transmembrane and intracellular domains from the γδ TCR with addition of an ET190L1-scFv/CD28 co-stimulatory molecule (ET019003 T cells) (96). ET019003 T cells were tested in preclinical studies followed by a phase I clinical trial in relapsed/refractory CD19^+^ B-cell leukemia and lymphoma (NCT04014894). So far, it was shown that these CAR-T cells presented a good safety profile and could induce rapid responses and durable CR in patients with relapsed or refractory DLBCL. Although, these results are preliminary and are limited to a small sample size, they offer new promising therapeutic strategies for patients with high-risk profiles. The spectrum of γδ T cells based IT in B-cell malignancies is summarized in the **Figure 3**.

## 5 Discussion and conclusion

Human γδ T cells present several specific characteristics that make them very attractive for their use in anti-cancer therapy in general and anti-lymphomatous in particular. Firstly, in contrast to αβ T cells, their anti-tumoral activity does not depend on mutational burden, thus rendering them efficient against tumors harboring few somatic mutations. Secondly, as they do not act dependently of MHC I-mediated Ag presentation, unlike CD8^+^ αβ T cells, they exhibit an anti-tumoral efficacy against tumors harboring a downregulation of surface MHC class I molecules. This characteristic is particularly well suited for the “off -the-shelf” allogenic cell therapy. Thirdly, they exhibit increased anti-tumoral activity due to their particular activation mechanisms present on both adaptive cells through the TCR signaling and innate cells through NK signaling (NKG2D, DNAM-1, NKp46, NKp44, NKp30). This is amplified by their low expression of killer inhibitor receptor.

Although Vδ2 T cell-based IT exhibited safety and good tolerance in patients, they also demonstrated limited success due to several reasons among which a highly variable capacity to recognize tumoral cells, functional instability, dysfunction or exhaustion of chronically activated Vδ2 T cells. Thus, innovative strategies were developed to improve tumoral cell recognition, promote durable persistence and circumvent exhaustion mechanisms involving Vδ2 but also Vδ1 T cells. In this context, engineered cells such as DOT and CAR-T offer very encouraging perspectives as well as combination of Vδ2 T cells with antibodies targeting ICP or neutralizing inhibitory cytokines to counteract immune suppression TME and exhaustion processes or transduction of selected high affinity Vγ9Vδ2 TCR into αβ T cells in order to induce a durable and memory-based response.

Several challenges remain, among them the difference of efficacy between cell engagers and ACT involving γδ T cells considering their logistical requirements and costs. Another important point to be considered is the justification for the selection of patients to be treated by such IT based on the identification of tumor Ag recognized by γδ T cells. All these issues will help to better understand, use and develop next-generation γδ T cell-based IT.

## Author contributions

LR, CF and MD contributed equally to the work of this review. CL participated in the writing. MP and CB wrote the review and supervised the work. All authors contributed to the article and approved the submitted version.
