# Correction: γδ T cells in immunotherapies for B-cell malignancies

**DOI:** 10.3389/fimmu.2026.1933915

**Published:** 2026-08-17

**Authors:** Léa Rimailho, Carla Faria, Marcin Domagala, Camille Laurent, Christine Bezombes, Mary Poupot

**Affiliations:** 1 Cancer Research Center of Toulouse (CRCT), UMR1037 Inserm-Univ. Toulouse III Paul Sabatier-ERL5294 CNRS, Toulouse, France; 2 Department of Pathology, Institut Universitaire du Cancer de Toulouse - Oncopôle, Toulouse, France

**Keywords:** immunotherapy, γδ T cells, lymphoma, leukemia, myeloma

An error in the main text was corrected to:

“However, other molecules such as CD226 (DNAX accessory molecule-1), adhesion molecules (LFA-1), CD3 or CD2, can also be involved in γδT cell activation and favor their immune responses. These different activation modes are summarized in **Figure 1**.”

**Figure 1** was revised.

**Figure 1:**
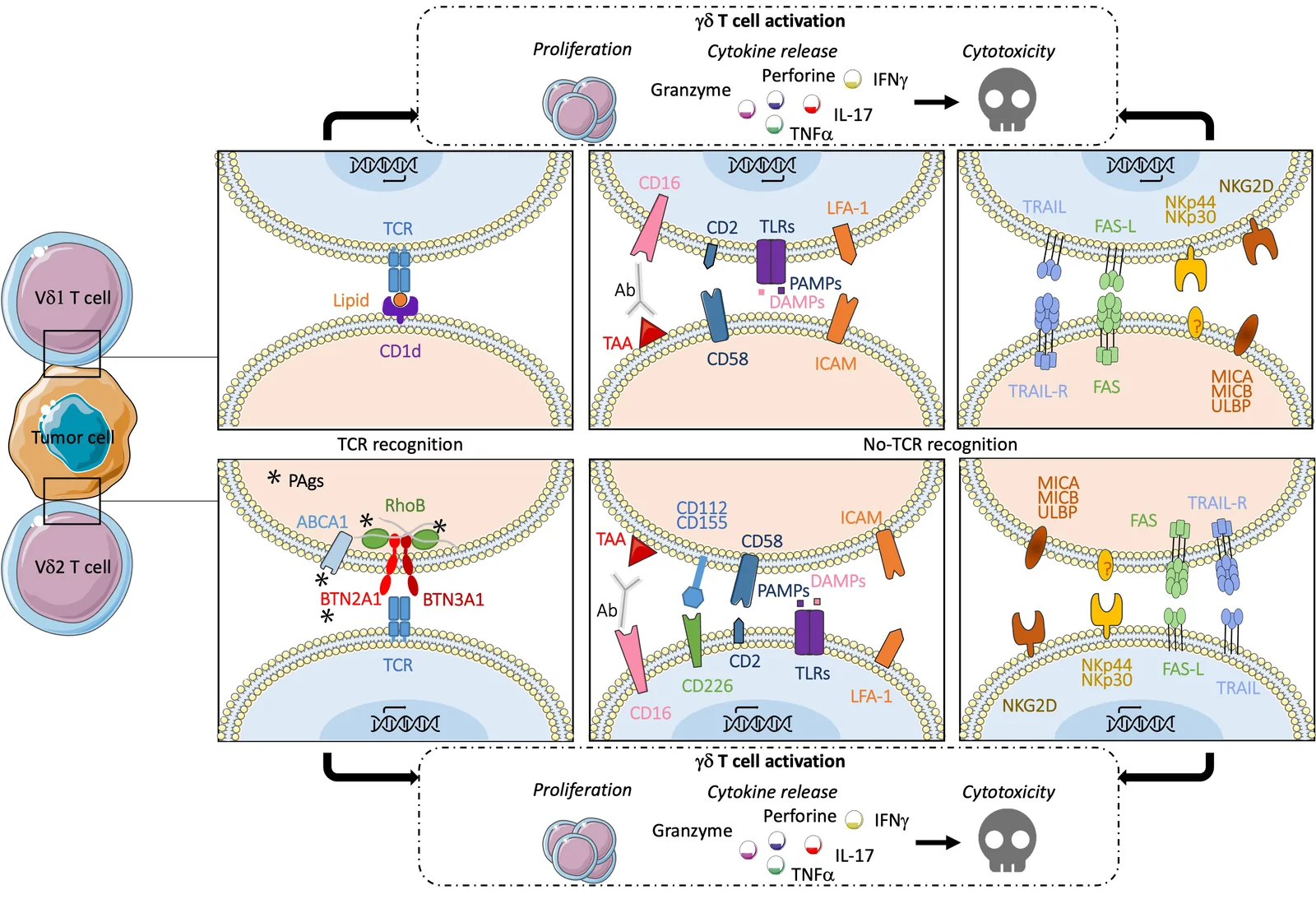
γδ T cell activation through different TCR-dependent and TCR-independent pathways leading to proliferation and/or cytokine release and/or cytotoxic signals.

And an addition was made to the **Acknowledgment** section:

“The authors warmly thank Théo Nouailhetas for his careful review, his insightful comments and for identifying errors that have significantly improved the manuscript.”

